# Fracture resistance of monolithic gradient zirconia crowns with different finish line designs and cement spaces

**DOI:** 10.1016/j.jtumed.2024.11.008

**Published:** 2024-11-29

**Authors:** Mohammed H. AbdElaziz, Mohamed F. Aldamaty, Elsayed Ali Omar, Adel A. Elbadawy, Sary Borzangy, Ahmed Y. Alqutaibi, Muhammad S. Zafar

**Affiliations:** a Substitutive Dental Sciences Department, College of Dentistry, Taibah University, Almadinah Almunawwarah 41311, KSA; b Department of Fixed Prosthodontics, Faculty of Dental Medicine, Al-Azhar University, Cairo 11651, Egypt; c Department of Restorative and Aesthetic Dentistry, College of Dentistry, Almaaqal University, Basrah, Iraq; d Prosthodontics Department, College of Dentistry, Taif University, Taif, KSA; e Prosthodontics Department, College of Dentistry, Ibb University, Ibb 70270, Yemen; f Department of Clinical Sciences, College of Dentistry, Ajman University, Ajman, United Arab Emirates; g Centre of Medical and Bio-allied Health Sciences Research, Ajman University, Ajman, United Arab Emirates; h School of Dentistry, Jordan University, Amman, Jordan; i Department of Dental Materials, Islamic International Dental College, Riphah International University, Islamabad 44000, Pakistan

**Keywords:** سماكة فراغ الاسمنت, خط نهاية الشطف, مقاومة الكسر, الزركونيا المتدرجة, خط نهاية حد السكين, خط نهاية الكتف, Cement space thickness, Chamfer finish line, Fracture resistance, Gradient zirconia, Knife-edge finish line, Shoulder finish line

## Abstract

**Objective:**

This study was aimed at assessing the effects of various finish line designs and cement gap thicknesses on the fracture resistance of gradient zirconia crowns.

**Methods:**

Sixty crowns were fabricated on stainless-steel dies with yttria multi-layered (YML) zirconia and categorized into three primary groups according to finish line type (knife-edge, chamfer, and rounded shoulder). Each group was further classified into two subgroups (n = 10) according to cement space thickness (50 or 80 μm). Optical impressions of the dies were acquired with an indirect laboratory scanner, and cement spaces (50 or 80 μm) were established for each finish line type in Exocad software. Subsequently, the zirconia crowns were milled, sintered, cemented onto their respective dies, thermomechanically aged, and subjected to loading until fracture. The data were statistically analyzed with one-way ANOVA and post hoc tests for pairwise comparisons. Additionally, two-way ANOVA was used to investigate interactions between two study variables.

**Results:**

No significant differences between chamfer and knife-edge patterns were observed, whereas the rounded shoulder pattern exhibited significantly higher failure load values. Similarly, no significant difference was observed between 50 μm and 80 μm cement space.

**Conclusions:**

Knife-edge margins with YML gradient zirconia crowns provide a reliable alternative to shoulder margins, particularly in minimally invasive preparations. A cement space of 80 μm rather than 50 μm is preferred for various finish line designs.

## الملخص

**أهداف البحث:**

يهدف البحث إلى تقييم تاثير تصميمات خط النهاية المختلفة وسماكات فجوة الاسمنت على مقاومة الكسر لتيجان الزركونيا المتدرجة.

**طرق البحث:**

تم تصنيع ستين تاجا على قوالب من الفولاذ المقاوم للصد باستخدام زركونيا اليتريا متعددة الطبقات وتصنيفها في ثلاث مجموعات رئيسية بناء على نوع خط النهاية (حد السكين، والشطف، والكتف المدور). تم تصنيف كل مجموعة لمجموعتين فرعيتين (العدد=10) على حسب سماكة فراغ الاسمنت (خمسون ميكرومتر، ثمانون ميكرومتر). تم الحصول على طبعات بصرية للقوالب باستخدام ماسح مختبري غير مباشر، وتم تحديد فراغات الاسمنت (خمسون وثمانون ميكرومتر) لكل نوع من خطوط النهاية من خلال استخدام برنامج اكسوكاد. بعد ذلك، تم تصنيع تيجان الزركونيا وتلبيدها ولصقها على قوالبها المعنية، وتم تعريضها للتقادم الحراري الميكانيكي، ثم تعريضها للتحميل حتى الكسر. خضعت البيانات المجمعة للتحليل الاحصائي من خلال استخدام تحليل التباين وحيد الاتجاه واختبارات المقارنات الزوجية. علاوة على ذلك، تم استخدام اختبار تحليل التباين ثنائي الاتجاه لدراسة تفاعل المتغيرين.

**النتائج:**

لم تلاحظ فروق مهمة بين نمطي الشطف وحد السكين. مع ذلك، سجلت مجموعة الكتف المدور قيم حمل فشل مرتفعة بشكل ملحوظ. وبالمثل، لم يكن هناك فرق مهم بين فراغ الاسمنت خمسين وثمانين ميكرومتر.

**الاستنتاجات:**

تمثل حواف حد السكين مع تيجان زركونيا اليتريا متعددة الطبقات المتدرجة بديلا موثوقا لحواف الكتف، خاصة في التحضيرات قليلة التدخل. يفضل فراغ الاسمنت ثمانين ميكرومتر على خمسين ميكرومتر لتصميمات خط النهاية المختلفة.

## Introduction

Increasing demand for aesthetically pleasing, metal-free restorations has led to the widespread use of all-ceramic systems.^1^, ^2^, ^3^, ^4^ However, one concern with these systems is the vulnerability of the interface between the zirconia core and the veneer, which can lead to chipping or cracking. To address this concern, monolithic zirconia restorations were developed, thereby eliminating the need for veneering and decreasing the risk of delamination.^5^, ^6^, ^7^, ^8^

Multi-layered zirconia has evolved over three generations (3Y, 4Y, and 5Y-TZP) to meet varying clinical requirements. These advancements offer a natural tooth shade gradient while maintaining flexural strength.^9^^,^^10^ Recent multi-layering technology has integrated the durability of high-strength 3Y-TZP in the dentin/body region with the high translucency of 5Y-TZP in the incisal/occlusal region. This innovation optimizes both stability and aesthetics; therefore, these materials may provide a promising option for restoring missing teeth.^11^^,^^12^

Vertical preparation techniques, such as knife-edge or feather-edge margins, have gained attention as a less invasive alternative to horizontal margins such as chamfers or shoulders.^13^ These techniques are particularly beneficial for teeth that have undergone periodontal treatment, teeth in young patients, endodontically treated teeth, and teeth with cervical caries.^14^^,^^15^ Although the clinical effects of vertical versus horizontal margins on periodontal health remain debated,^16^ histological studies have suggested that margin design does not significantly influence periodontal health.^17^ Furthermore, knife-edge margins have been demonstrated to have similar clinical outcomes to other designs, while providing benefits of smaller marginal openings and less invasive preparation.^18^

Studies comparing finish line designs, such as chamfers, shoulders with acute axio-gingival line angles, and shoulders with rounded axio-gingival line angles, have shown notably diminished strength of chamfer designs.^19^, ^20^, ^21^ However, other research has indicated no significant difference in fracture strength between crowns with chamfer or knife-edge finish lines, thus suggesting that invasive finish line preparations might not always be necessary to achieve proper attachment of ceramic crowns to enamel.^22^

One important consideration for the long-term success of ceramic restorations is internal adaptation. Insufficient adaptation can compromise fracture resistance, particularly when excess cement leads to residual stresses due to cyclic loading. These stresses, caused by viscoelastic deformation, can damage veneering porcelain, and lead to chipping and failure in zirconia-based restorations.^23^ Despite advancements in 3D-printed zirconia,^24^, ^25^, ^26^ milled zirconia remains the most frequently used material for critical dental restorations such as crowns and bridges.

Aging caused by thermal fluctuations in the oral environment can adversely affect the mechanical properties of ceramic restorations. Thermal stress, simulated through thermocycling, has been shown to degrade translucent monolithic zirconia. This degradation may be attributable to low-temperature degradation and monoclinic phase transformation. Therefore, materials are recommended to undergo thermocycling testing.^27^ Moreover, the success of ceramic restorations relies on factors such as fracture strength, marginal fit, and esthetics. These factors are influenced by preparation design, material properties, cement space thickness, and the choice of luting agent.^28^

Limited research has examined the combined effects of finish line design and cement space thickness on yttria multi-layered (YML) zirconia crowns. Therefore, this study was aimed at assessing how various finish line designs and cement space thicknesses affect the fracture resistance of YML zirconia crowns. The null hypothesis was that neither variable would significantly influence fracture resistance, whereas the alternative hypothesis was that both finish line design and cement space thickness would have notable effects on crowns’ fracture resistance.

## Materials and Methods

### Metal die manufacturing

**Figure 1:**
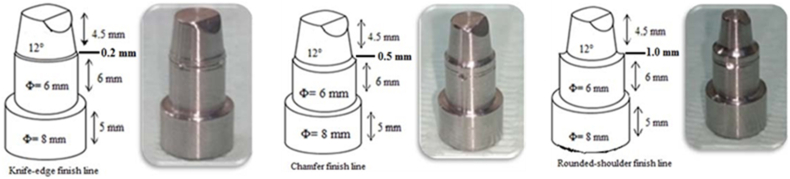
Diagram for master die fabrication.

Three stainless-steel dies were precisely machined to replicate the preparation of maxillary first premolars in an experimental setting. Each die was designed to have a height of 4.5 mm and a flat occlusal surface, with a total convergence angle of 12°.^29^ Additionally, to ensure accurate realignment of the crowns during subsequent measurements, a beveled surface was carefully crafted at one side of each die at the occlusal-axial line angle. Notably, the three dies had distinct finish lines: a knife-edge finish line (K) with a thickness measuring 0.2 mm, a chamfer finish line (C) with a thickness of 0.5 mm, or a rounded shoulder finish line (S) with a thickness of 1.0 mm Figure (1).

### Power test analysis

The G∗Power statistical analysis program (version 3.1.9.4) was used for sample size calculation. A total sample size of 36 participants (12 per group, subdivided into six per subgroup, n = 6) was considered sufficient to detect a large effect size (f) of 0.69, with an actual power (1 - β error) of 0.95 (95 %) and a significance level (α error) of 0.05 (5 %) for a two-sided hypothesis test. However, a larger sample size (n = 10) was used, thus resulting in a post-study power of 1, or 100 % (Table 3). A total of 60 YML monolithic gradient zirconia crowns (Katana, Kuraray Noritake Dental, Inc.) were divided into three primary groups of 20 crowns each, according to finish line design: group C, group K, and group S. Furthermore, each primary group was divided into two subgroups, comprising ten crowns each, according to the thickness of the cement space: 50 μm^30^ or 80 μm.^31^

### Specimen fabrication

All crowns were fabricated with a five-axis milling machine (Roland DWX-51D, Roland DGA Corp, California). The metal dies were sprayed with scan spray (Shera GMBH, Lemförde, Germany), and optical impressions were taken with an indirect laboratory scanner (Identica Hybrid, Medit, Seoul, Korea). The scan data were imported into CAD software (Exocad 3.0 Galway GmbH, Darmstadt, Germany) for progressive crown design, and a cement space of 50 μm or 80 μm was applied to all finish line design variations. All zirconia crowns were milled from YML material (Katana, Kuraray, Noritake, Inc.) with an occlusal thickness of 2 mm and an axial wall thickness of 1 mm, incorporating the three finish line designs (K, C, and S). Subsequently, the crowns were sintered at a temperature of 1450 °C with 2 h' holding time, according to the manufacturer's instructions. Each sintered crown was examined on its respective die and assigned a unique serial number based on the grouping.

### Cementation procedures

**Figure 2:**
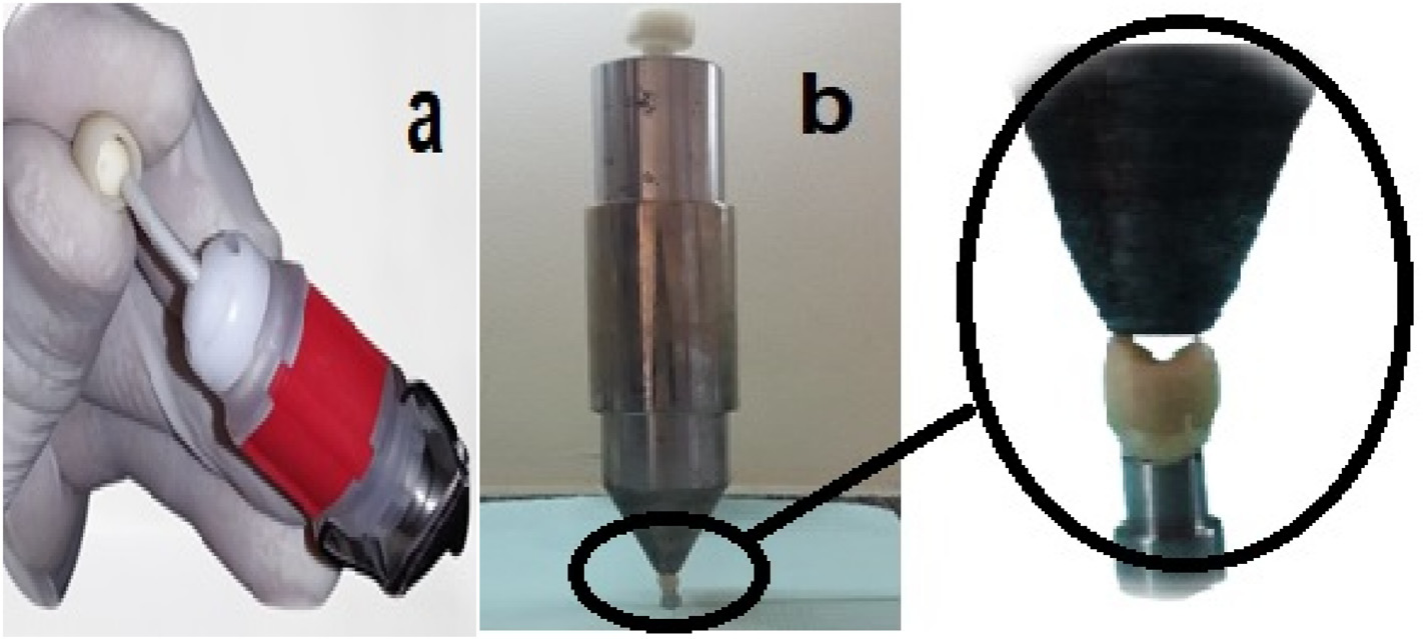
a: Dispensation of cement into the fitting surface. b: Cementation under static load.

**Figure 3:**
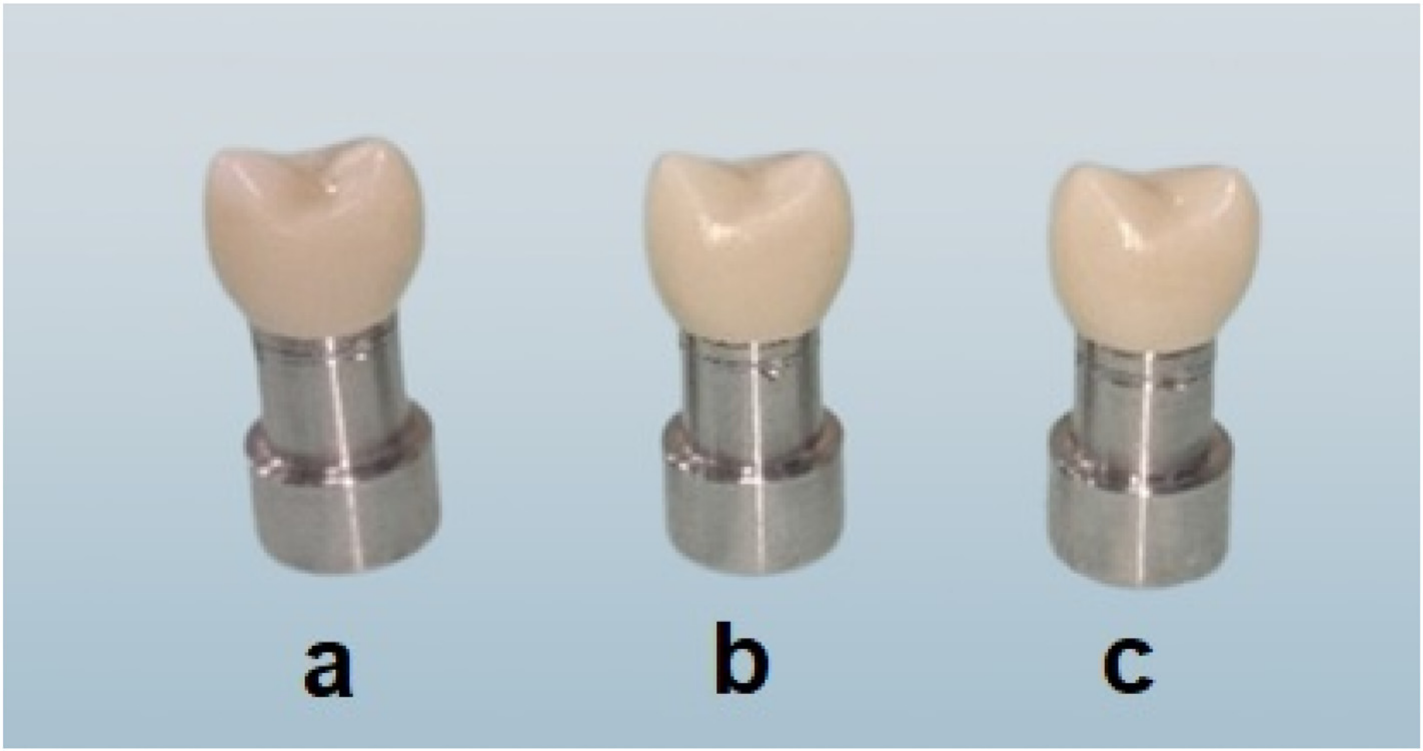
Cemented crowns on their corresponding dies. a: Knife edge. b: Chamfer. c: Rounded shoulder.

The crowns were cemented by a single operator using traditional glass ionomer cement (KetacTM Cem, 3M ESPE) (Figure 2a). Initially, the crowns were positioned with finger pressure, and a specialized cementing device was then used to apply an axial load (Figure 2b). Subsequently, a static load of 50 N was applied for 6 min to replicate the bite force experienced during clinical placement and to allow the cement to solidify.^32^ The excess cement was removed with a scaler. The cemented specimens were then incubated for 24 h at 37 °C in deionized distilled water (Figure 3).

### Testing conditions

After cementation, the crowns were exposed to thermal cycling and subjected to chewing simulation. Thermal aging was performed with a THE 1100 Thermocycler (SD Mechatronik GMBH in Miesbacher, Feldkirchen-Westerham, Germany) in 10,000 cycles. The distilled water level in the system was verified daily and adjusted if necessary, without affecting the water bath temperature. The water bath temperature range was 5–55 °C, and the bath/dwell cycles lasted 10–60 s.^27^^,^^33^ After undergoing thermocycling, the specimens were subjected to aging in an ACH-09075DC-T chewing simulator manufactured by ADTECH Technology Co., Ltd. (Germany). A stainless-steel antagonist stylus was used to transfer force to the center of the occlusal surface. The parameters for this process are listed in Table 1. The simulation involved 118,000 cycles, equivalent to 1 year of use.^34^

**Table 1:** Chewing simulator parameters.

Bath temperature: 5/55 °C	Dwell time: 60 s
Horizontal movement: 1 mm	Vertical movement: 3 mm
Forward speed: 90 mm/s	Rising speed: 90 mm/s
Backward speed: 40 mm/s	Descending speed: 40 mm/s
Weight per sample: 10 kg (98N)	Cycle frequency: 1.6 Hz
Torque: 2.4 Nm	

### Testing procedures

**Figure 4:**
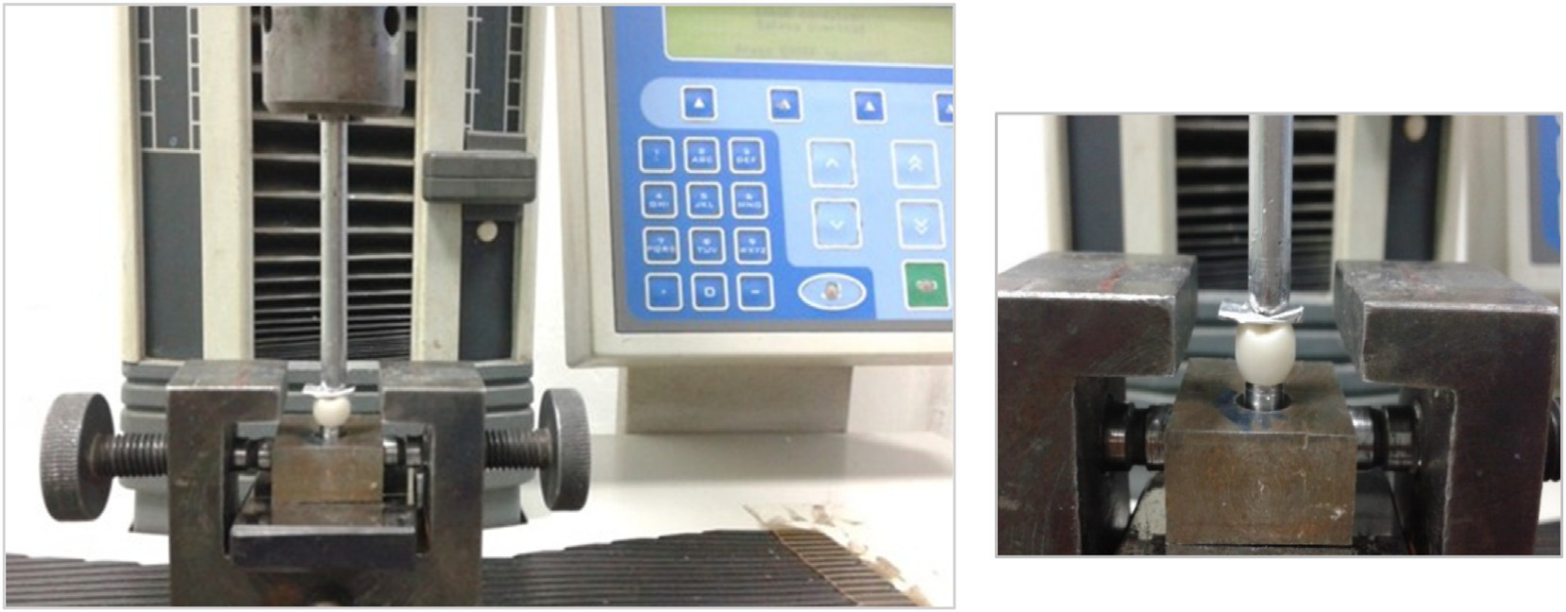
Loading until fracture with the universal testing machine.

The cemented crowns underwent fracture testing with a universal testing machine (Model LRX-Plus, Lloyd Instruments, Fareham, UK) equipped with a load cell (5 kN) and a semi-spherical head (3.8 mm diameter) on the loading piston. The piston was placed directly above the occlusal surface of each crown sample and oriented centrally toward the central groove, while contacting the triangular ridges of both the buccal and lingual cusps. A vertical force was gradually exerted at a rate of 1.0 mm/min until failure. To achieve a consistent distribution of stress and minimize the occurrence of localized force peaks, we positioned an interlayer of tin foil between the load piston and the specimen. The occurrence of failure was identified according to an audible cracking sound, which was subsequently corroborated by a substantial decline in the load-deflection curve. The data were captured and analyzed in Nexygen-MT software from Lloyd Instruments (Figure 4).

After the fracture resistance tests, the mode of fracture in the fractured specimens was visually examined with magnifying lenses and classified according to Burke's system^35^ as follows: class I, crack or a minimal fracture in the crown; class II, loss of restoration (less than half the crown); class III, fracture across the midline, resulting in displacement or loss of half the crown; class IV, loss of more than half the crown; and class V, severe fracture involving both the tooth and crown. To ensure reliability, assessments were conducted by the same investigator 1 week apart and by an independent examiner. The intra-observer reliability (1.0) and inter-observer reliability (0.96) were excellent.

### Statistical analysis

Data were analyzed in the SPSS v20.0 statistical software package (IBM SPSS Statistics for Windows, Version 20.0; Armonk, NY, USA). The mean and standard deviation were recorded to summarize numerical values, along with confidence intervals and ranges. Because the data were normally distributed, as confirmed by Kolmogorov–Smirnov and Shapiro tests, the groups were analyzed with one-way ANOVA. Post hoc pairwise comparisons were conducted with Bonferroni's test. Intra-group comparisons were performed with independent t-tests. Interactions between study variables were analyzed with two-way ANOVA. The threshold for statistical significance was P ≤ 0.05.

## Results

### Fracture resistance

#### Cement Space Thickness of 50 μm

The highest mean fracture resistance was observed for the S group (1935.98 ± 279.9 N), which was followed by the C group (1678.30 ± 277.12 N), whereas the lowest value was recorded in the K group (1474.98 ± 297.35 N). The fracture resistance was significantly greater in the S group than the P group (P = 0.005). Post hoc tests revealed that the results did not significantly differ between the chamfer group and the other groups.

#### Cement Space Thickness of 80 μm

The highest mean fracture resistance was observed in the S group (2120.93 ± 214.83 N), which was followed by the C group (1760.33 ± 205.91 N), whereas the lowest value was observed in the K group (1564.43 ± 278.45 N). The fracture resistance was significantly greater in the S group than the other two groups (P = 0.000). Post hoc tests revealed that the results for the C and K groups did not significantly differ (Table 2).

**Table 2:** Descriptive statistics of fracture resistance (Newtons), between groups (ANOVA) and intragroup comparisons for cement space thicknesses of 50 μm and 80 μm (independent t-test).

**Group**	50 μm		80 μm		Both thicknesses		P-value (between thicknesses)
	Mean	SD	Mean	SD	Mean	SD	
Shoulder group (S)	1935.98^a^	279.90	2120.93^a^	214.83	2028.45^x^	260.72	0.115 ns
Chamfer group (C)	1678.30^a,b^	277.12	1760.33^b^	205.91	1719.32^y^	241.31	0.462 ns
Knife-edge group (K)	1474.98^b^	297.35	1564.43^b^	278.45	1519.71^y^	284.10	0.496 ns
All groups	1696.42	335.26	1815.23	326.34	1755.82	333.44	0.084 ns
P-value between finish lines	0.005∗		0.000∗		0.000∗		

Significance level: significant (∗P ≤ 0.05), non-significant (ns); μm, micrometer; SD, standard deviation.

Post hoc test: values with the same superscript letters (^a,b,x,y^) did not significantly differ.

**Table 3:** Two-way ANOVA for the interaction of variables and power of the study.

**Source**	Sum of squares for type III	df	Mean square	F	P-value	Observed power
Finish line	2628237.940	2	1314118.97	19.25	0.000∗	1.000
Cement space thickness	211732.489	1	211732.49	3.10	0.084 ns	0.409
Finish line × cement space thickness	32940.359	2	16470.18	0.24	0.786 ns	0.086

Significance level: significant (∗P ≤ 0.05), non-significant (ns); df, degrees of freedom; F, Fisher's F ratio.

### Effects of cement space on fracture resistance

In the S group, no significant difference in the recorded values at 50 μm (1935.98 ± 279.9 N) and 80 μm (2120.93 ± 214.83 N) (P = 0.115) was observed. Likewise, in the C group, no significant difference (P = 0.462) in fracture resistance was observed between the 50 μm (1678.30 ± 277.12 N) and 80 μm (1760.33 ± 205.91 N) specimens. Moreover, in the K group, no significant difference was observed between the values recorded at 50 μm (1474.98 ± 297.35 N) and 80 μm (1564.43 ± 278.45 N) (P = 0.496) (Table 2).

### Interaction of variables and power of the study

For any cement space thickness, the highest average fracture resistance value (2028.45 ± 260.72 N) was observed for the S group, which statistically significantly differed from those in the other groups (P = 0.000). The C group followed, with an average value of 1719.32 ± 241.31 N, whereas the K group had the lowest average fracture resistance value (1519.71 ± 284.1 N). According to the post hoc tests, no statistically significant difference (P > 0.05) was observed between the C and K groups. For any finish line design, the 50 μm cement space thickness group had an average fracture resistance value (1696.42 ± 335.26 N) that did not significantly differ (P = 0.084) from that in the 80 μm cement space thickness group (1815.23 ± 326.34 N). Two-way ANOVA demonstrated no significant interaction effect for both study variables (finish line and cement space thickness) (P = 0.786). The power of this study (1-β error) was 1, i.e., 100 % (Table 3).

### Mode of fracture

**Figure 5:**
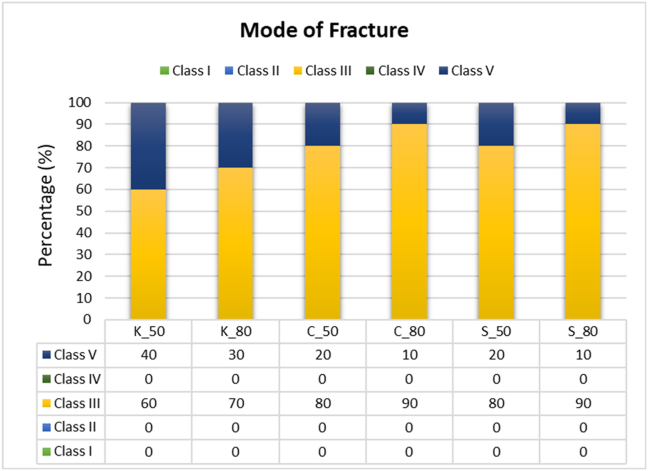
Percentages of each fracture mode for each tested group.

**Figure 6:**
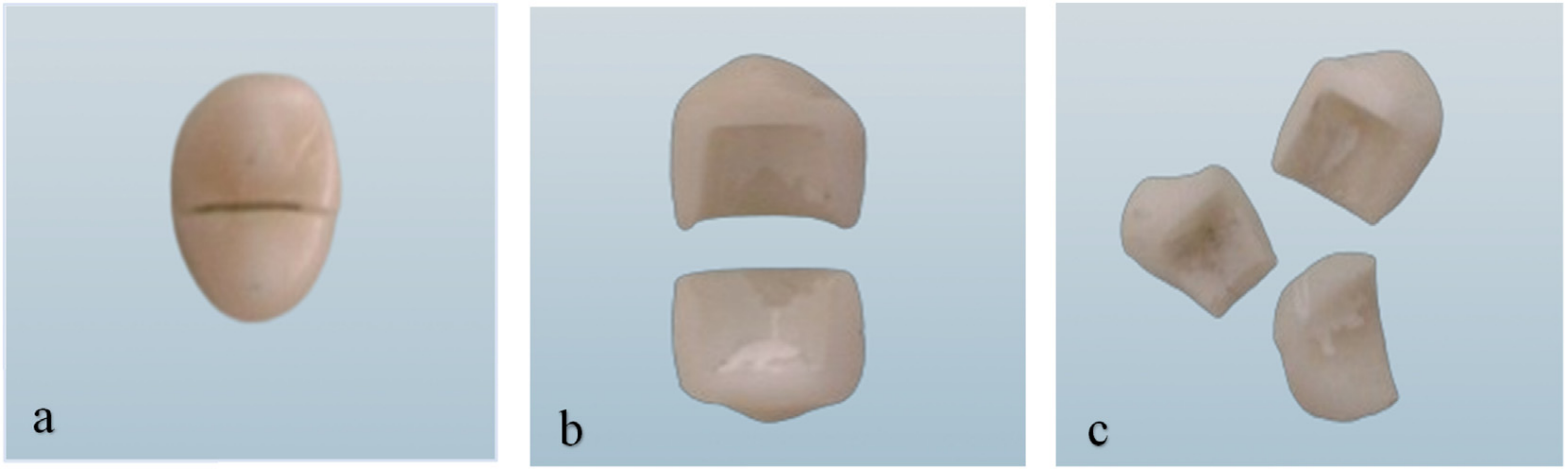
Fracture mode, according to Burke's classification system; a and b correspond to fractures across the midline (class III), whereas c corresponds to severe fracture (class V).

The collected specimens exhibiting fractures were visually inspected to determine the mode of fracture (Figure 5). Fractures were classified with Burke's system.^35^ The frequency (n) and percentage (%) of each fracture mode across the groups are presented in Figure 6.

## Discussion

This study investigated the effects of various crown preparation designs and cement space gaps on the fracture resistance of YML zirconia crowns. After testing of the fracture resistance of gradient zirconia crowns with various finish line designs and cement spaces, the null hypothesis was partially rejected. With a 50 μm thickness, the highest fracture resistance was found in the S group (1935.98 N), which significantly outperformed the K group (P = 0.005), whereas the C group showed no significant differences with respect to the other groups. With an 80 μm thickness, the S group again exhibited the highest fracture resistance (2120.93 N), which was significantly greater than that in the C and K groups (P = 0.000). The effect of cement space was not significant within any group, and no notable differences in fracture resistance were observed between 50 μm and 80 μm thicknesses across all groups.

We used monolithic gradient zirconia to address the issue of delamination frequently associated with conventional zirconia restorations, by milling fully contoured restorations with anatomical precision, thereby eliminating the requirement for veneering porcelain, in accordance with Ban's study.^6^ In the present investigation, as in several previous experiments, we used a stainless-steel die as an abutment.^14^^,^^29^ Metal dies have several advantages, including standardized preparation, and minimal wear during the production and measuring procedures.

To replicate the crown preparation process, we machined the metal dies. The preparation specifications included a height of 4.5 mm, ensuring a minimum occluso-cervical dimension of 3.0 mm, and a total occlusal convergence of 12° for premolars, as recommended in previous studies.^14^^,^^29^^,^^36^ For the bonding of ceramic crowns to prepared teeth, two options for the finish line are available: chamfer or shoulder. A finish line depth ranging from 0.5 to 1.0 mm has been recommended.^36^ Recently, a new approach called the knife-edge finish line has been introduced for zirconia crowns. This method is less invasive than the other methods and has shown good clinical results, because minimal tooth reduction helps to preserve more of the natural tooth structure while still providing adequate support for the crown.^18^

Comlekoglu et al.^17^ have reported that a knife-edge finish line leads to smaller marginal openings. However, the authors still recommended using shoulder and mini chamfer designs, because of their potential to create a wedging effect at the margin and possibly increase the size of the marginal bulk. Both biological and technical factors support this recommendation. Nevertheless, the recommendation to refrain from using knife-edge margins has not been empirically supported by a clinical investigation by Poggio et al.,^18^ in which the knife-edge design had comparable clinical outcomes to other margin designs while requiring more conservative preparation. Furthermore, this recommendation for using mini-chamfer and shoulder designs is contradicted by findings from another study.^15^ Histological evidence has indicated no discernible variation in periodontal health across various margin design patterns. Furthermore, knife-edge margins have not been found to affect gingival health in a cohort of individuals with periodontal disease.^16^

In the current study, thermomechanical aging was applied to specimens to simulate approximately 1 year of clinical service. Thermomechanical aging is a helpful approach for estimating the clinical performance of restorations by reproducing temperature variations in the oral environment; the same processes have been performed in previous investigations.^37^^,^^38^

Regardless of the cement space, the rounded shoulder group exhibited the highest fracture resistance, possibly because of augmented material thickness. These findings align with those from previous studies^19^^,^^20^ also reporting significantly greater strength in crowns with a shoulder finish rather than a chamfer finish. In contrast, Beuer et al.^14^ have reported no statistically significant difference between crowns positioned over preparations without a shoulder and those placed on preparations with a shoulder. Hence, Goodacre et al.^36^ have proposed using shoulder finish lines for ceramic crowns in which etching and bonding procedures are not required. Additionally, the authors have suggested that both shoulder and chamfer preparations may be used for ceramic crowns. We observed no significant differences in the fracture strengths of crowns affixed to dies with knife-edge versus chamfer finish lines, in agreement with the findings of Cortellini et al.^22^

Regardless of the margin design, an 80 μm cement space provided higher fracture resistance than 50 μm cement space, although the difference was statistically non-significant. These findings align with previous studies.^31^^,^^39^ These results may be explained from a biomechanical perspective by considering the fit and stress distribution at the cement interface. A larger cement space, such as 80 μm, allows for better adaptation of the restoration to the underlying tooth structure, thus ensuring a more uniform distribution of occlusal forces and therefore minimizing localized stress concentration that could lead to early fractures. In contrast, a 50 μm cement space might lead to less optimal seating of the crown and uneven force distribution, thus potentially compromising the structural integrity of the restoration. Consequently, the enhanced adaptation provided by the 80 μm space contributes to greater fracture resistance.^31^^,^^39^ Furthermore, a cement space thickness of 80 μm allows for better seepage of excess cement in the cervical area compared with the occlusal area during crown seating. Therefore, a thinner cement film thickness effectively decreases hydraulic pressure, promotes even distribution of cement, and ultimately helps avoid any discrepancies in crown seating.^39^ Furthermore, with an 80 μm cement space thickness, an increased bonding interface consequently augments micromechanical interlocking, bonding action, and fracture strength.^40^ In addition, Liu et al.^41^ have reported that a greater thickness of luting material enables the crown to flex more, thereby increasing tensile stress within the core. In contrast, a thinner layer of cement limits the amount of mechanical energy that the luting material can absorb, and the stress levels in the crown therefore increase. These findings indicate that cement space thickness is not as influential as the loading conditions or the properties of the cement itself.

After consideration of all relevant factors, the average breaking loads of all tested preparation designs exceeded the clinically necessary strength threshold for zirconia, which is 1000 N.^14^ The load value of 1000 N was determined according to empirical evidence that the strength of zirconia decreases by as much as 50 % after exposure to the conditions of the oral cavity. Additionally, the average masticatory force in the posterior region is assumed to be 300 N, and a safety margin of 200 N is incorporated to account for potential variations and uncertainties.^14^ The knife-edge group exhibited the lowest fracture resistance in the current investigation, at 1519.71 N. However, this value is more than threefold higher than the clinically acceptable threshold required for the posterior region, which is typically within the range of 300–500 N.

Comparison of fracture modes indicated that 47 of 60 crowns (78.33 %) showed fracture through the midline (class III), whereas only 13 crowns (21.66 %) showed severe or catastrophic fracture of the crown (class V). The high percentage of crowns (78.33 %) that fractured through midline fissure might have been be due to the universal testing machine's static loading exerted axially on the occlusal surface; the crowns fractured at the thinnest part (fissure), where the weakest point was located.

The findings of the present study provide valuable insights for clinicians selecting preparation designs and cement spaces to enhance the durability of dental restorations. Knife-edge margins are recommended as a reliable option for YML gradient zirconia crowns, offering promising outcomes, particularly in cases requiring minimally invasive preparation, thus providing a viable alternative to shoulder margins. Additionally, a cement space thickness of 80 μm is preferred over 50 μm for various finish line designs, because it ensures enhanced fit and performance without compromising the restoration's fracture resistance.

One of this study's limitations was the use of metal dies instead of natural teeth. Metal dies, although providing a uniform and controlled testing environment, do not perfectly replicate the complex structure and varying properties of natural teeth. Natural teeth have diverse compositions, including enamel, dentin, and pulp, each with distinct mechanical and physical characteristics that influence dental restorations' performance and fracture resistance. Consequently, results obtained from metal dies might not fully capture the clinical behavior of materials applied to natural teeth, thus potentially limiting the applicability and generalizability of our findings to real-world dental scenarios. However, the use of metal dies is justified in research, because it enables standardization and consistency in fracture resistance measurement. This standardization ensures that the tested variables are isolated and controlled, thereby providing more reliable and reproducible results, which are crucial in the initial stages of material testing and development.^29^

## Conclusions

The following conclusions were drawn from the present study:

1. Knife-edge margins are recommended as a reliable option for YML gradient zirconia crowns, thus providing secure outcomes, particularly in cases requiring minimally invasive preparation, and serving as a viable alternative to shoulder margins.
2. A cement space thickness of 80 μm is preferred over 50 μm for various finish line designs, thereby ensuring enhanced fit and performance.

## Source of funding

This research received no external funding

## Ethics approval and consent to participate

This article does not contain any studies in human participants performed by any of the authors.

## Authors contributions

Conceptualization, M.H.A., MFA, EAO, AAE; methodology, AAE, SB; validation, MFA, EAO, AAE, SB; formal analysis, M.H.A., MFA, EAO, AAE, SB; investigation, M.H.A., MFA; resources, SB; data curation, M.H.A., MFA, SB, MSZ, and AYA; writing—original draft preparation, M.H.A., MSZ, and AYA; writing—review and editing, M.H.A., MFA, SB, MSZ, and AYA; visualization, MSZ, and AYA; supervision, M.H.A.; project administration, M.H.A. and MFA. All authors have read and agreed to the published version of the manuscript. All authors have critically reviewed and approved the final draft and are responsible for the content and similarity index of the manuscript.

## Conflict of interest

The authors declare no conflict of interest.
